# Daily cardiac autonomic responses during the Tour de France in a male professional cyclist

**DOI:** 10.3389/fnins.2023.1221957

**Published:** 2024-01-08

**Authors:** Nicolas Bourdillon, Samuel Bellenoue, Laurent Schmitt, Grégoire P. Millet

**Affiliations:** ^1^Institute of Sport Sciences (ISSUL), University of Lausanne, Lausanne, Switzerland; ^2^COFIDIS Pro Cycling Team, Villeneuve d'Ascq, France; ^3^National Centre of Nordic-Ski, Research and Performance, Premanon, France

**Keywords:** cycling, heart rate variability, performance, Tour de France, fatigue, elite

## Abstract

**Background:**

Heart rate variability (HRV) is a common means of monitoring responses to training, yet in professional cycling, one may question its usefulness, particularly during multi-day competitions such as Grand Tours.

**Objectives:**

This study aims to report and analyze HRV responses in a male professional cyclist over a season, including the Tour de France.

**Methods:**

A professional cyclist recorded resting and exercise inter-beat intervals during 5 months, comprising a training period with two altitude sojourns and two competition blocks, including the Tour de France. Resting recordings lasted 5 min in the supine position and were used for computation of mean heart rate (HR), root mean square of the successive differences (RMSSDs), and power in the low- and high-frequency bands (LF and HF, respectively). Training load quantification was based on recorded HR during exercise and expressed as training impulses (TRIMPSs).

**Results:**

LF (3,319 ± 2,819 vs. 1,097 ± 1,657 ms^2^), HF (3,590 ± 1858 vs. 1,267 ± 1,683 ms^2^), and RMSSD (96 ± 26 vs. 46 ± 30 ms) were higher and HR (47 ± 4 vs. 54 ± 2 bpm) was lower during the training period when compared to the two competition blocks. The coefficient of variation (CV) was significantly lower during the training period than during the two competition blocks for RMSSD (26 vs. 72%), LF (85 vs. 160%), and HF (58 vs. 141%).

**Discussion:**

The present study confirms that monitoring daily HRV responses during training periods is valuable in professional cycling, but questions its usefulness during the Tour de France. Moreover, the previous suggestion that CV in RMSSD would help to predict poor performance was not confirmed in a professional cyclist.

## Introduction

Heart rate variability (HRV) is a means of assessing the impact of training sessions on athletes’ homeostasis that is commonly used in endurance sports (Manzi et al., 2009; Plews et al., 2012; Schmitt et al., 2021). It allows us to determine whether there is an autonomic misbalance (Schmitt et al., 2015) and whether an athlete is at risk of functional, non-functional overreaching, or overtraining syndrome (Meeusen et al., 2013).

Previous studies evidenced that monitoring HRV during training resulted in performance improvements because the training load could be adapted on a daily basis to what the athletes could bear (Schmitt et al., 2018). Moreover, it was previously proposed that the analysis of the day-to-day variation in HRV parameters would be valuable for diagnosing non-functional overreaching (Plews et al., 2012). However, its usefulness during multi-day competitions, such as during professional cycling Grand Tours, remains unclear.

In professional cycling, the 3-week Tour de France is the most prestigious event and is highly challenging for human homeostasis (Lucia et al., 2003). Yet, studies analysing HRV responses during competitions in professional cycling are scarce (Earnest et al., 2004). Even though a coach cannot manipulate the load during competition, these data are paramount to better understanding the impact of professional competition on athletes’ homeostasis and helping optimize the recovery periods between competitions.

The present case study aimed to analyze the daily cardiac autonomic responses as well as training loads of a professional cyclist involved in multiple races during the 2020 season, including the Tour de France.

## Methods

### Participant

A top professional cyclist (28 years old, height 173 cm, weight 57 kg; top 10 on the Tour de France and Vuelta España) recorded beat-to-beat heart rate in the supine position for 5 min, resulting in a total of 86 recordings over the 5 months. He gave written informed consent for data recording and utilization in the context of training and research. This project was approved by the Necker Hospital Ethics Committee (Paris, France).

### Experimental design

HRV recordings were performed during pre-season preparation (training block from 14 May to 5 August 2020) and competition periods, which consisted of two blocks.

Pre-season training comprised two altitude camps. The first (2 June–16 June) was based on a live-high (LHTL, 2700 m, 16 h/day) train-low (1,150 m) protocol using a normobaric hypoxic chamber, whereas the second camp (14 July–2 August) was performed on a live-high train-high protocol (LHTH, 2000 m) in the mountains.

The competition block #1 spanned from 6 August to 23 August 2020 and included participation in *Mont Ventoux Dénivelé Challenge* (186 km, 4,100 m elevation gain, 6 August, ranked top 5), *Tour de l’Ain* (3-stage race, 140, 141, and 145 km, rolling and mountain stages, 7–9 August, ranked top 8), *Critérium du Dauphiné* (5-stage race, 219, 181, 176, 173, and 154 km, mountain stages, 12–16 August, ranked top 3), and French Championship (238 km, flat, 23 August, ranked top 12).

The competition block #2 consisted of the *Tour de France* (21-stage race including flat, rolling, mountain, and time-trial stages, 29 August–20 September, ranked top 12).

### Procedures

#### Heart rate variability

HRV recordings were performed in the supine position after wake-up, while fasting, with an empty bladder, and in a quiet environment (Bourdillon et al., 2017). A heart rate monitor (Polar H10, Kempele, Finland), connected via Bluetooth to a smartphone application (inCORPUS®, v2.4.6, be.care S.A., Renens, Switzerland), was used for RR interval storage.

Out of the 5-min period, the last 4 min were analyzed (Bourdillon et al., 2017). Ectopic beats were compensated to calculate normal-to-normal intervals using visual and automated inspections [MATLAB® (R2019a, MathWorks, Natick, MA, USA)]. Mean HR, root mean square of the successive differences (RMSSDs), spectral power density in the low-frequency (LF, 0.04–0.15 Hz), and high-frequency bands (HF, 0.15–0.40 Hz) in ms^2^ were computed (Task Force, 1996).

#### Training/competition load

During training and competition, the athlete wore a heart rate monitor to record the beat-to-beat heart rate. The training load was computed using Banister’s TRIMPS method (Banister and Calvert, 1980).


$$\mathrm{TRIMPS}=\mathrm{HRratio}\times \mathrm{time}\;\mathrm{interval}\times \exp^{1.92\times \mathrm{HRratio}}$$

where HRratio is (HR – HRmin) divided by (HRmax – HR). HRmin was the lowest heart rate found during supine recordings (40 bpm), while HRmax was the highest heart rate found at maximal exercise (198 bpm). TRIMPSs were quantified for each training/competition session and represent the load put on the cardiovascular system. It is a useful and widespread tool.

### Statistics

All data are presented as mean ± standard deviation (SD). The normal distribution of the data was checked using the Shapiro–Wilk test. Comparison of means was performed using the student’s t-test; comparison of SDs was performed using the χ^2^ test; and comparison of coefficients of variation (CV) was performed using the permutation test. All analyses were performed using MATLAB® (R2019a, MathWorks, Natick, MA, USA).

## Results

**Figure 1:**
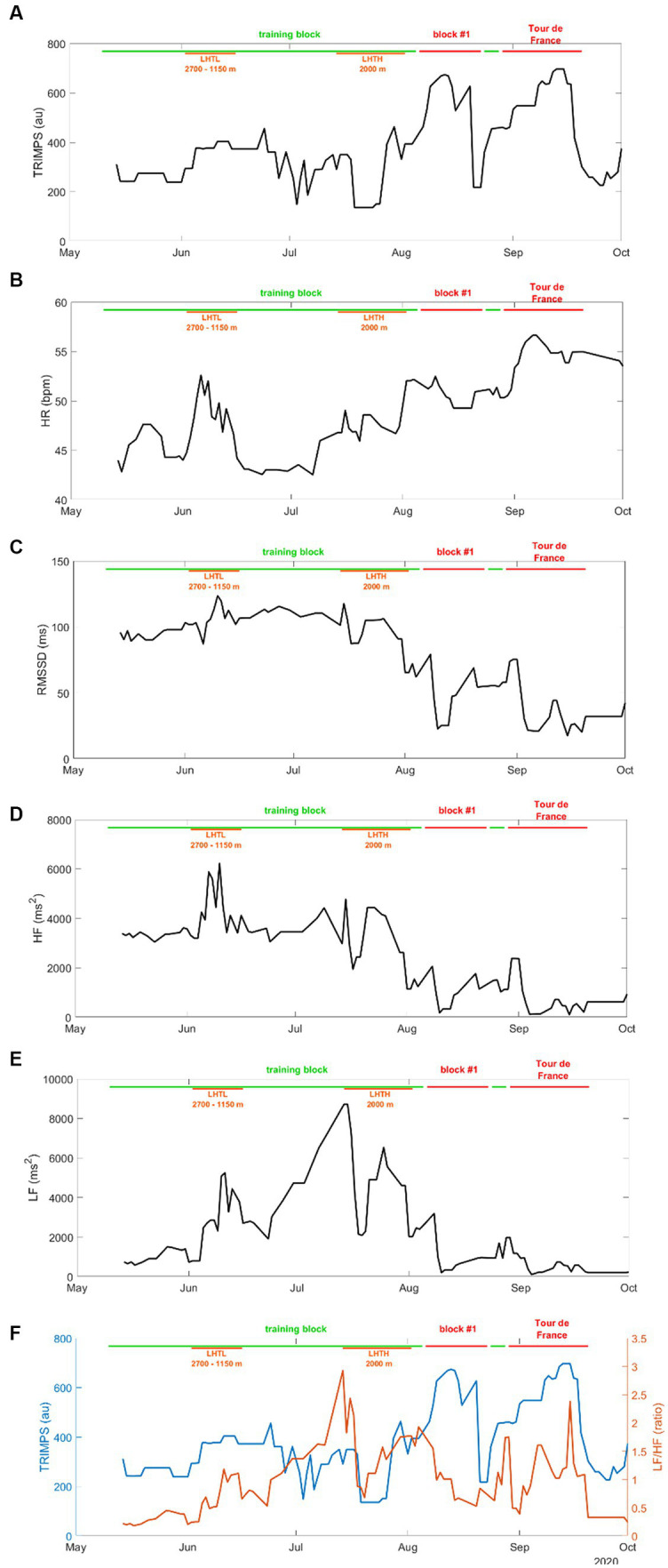
**(A)** TRIMPS (au) of training or competition. **(B)** HR (bpm) in the supine position. **(C)** RMSSD (ms) in the supine position. **(D)** HF (ms^2^) in the supine position. **(E)** LF (ms^2^) in the supine position. **(F)** TRIMPS and LF/HF ratio superimposed. All panels between 14 May and 01 October 2020.

HRV parameters and TRIMPS values across the season are presented in Figure 1. TRIMPSs were significantly higher during the competition periods than during training (Figure 1A). Supine HR temporarily increased during training and then continuously and significantly increased during competition (Figure 1B). RMSSD and HF (Figures 1C,D) were steady during training and significantly decreased during competition, whereas LF progressively increased during training and largely decreased during competition (Figure 1E).

**Figure 2:**
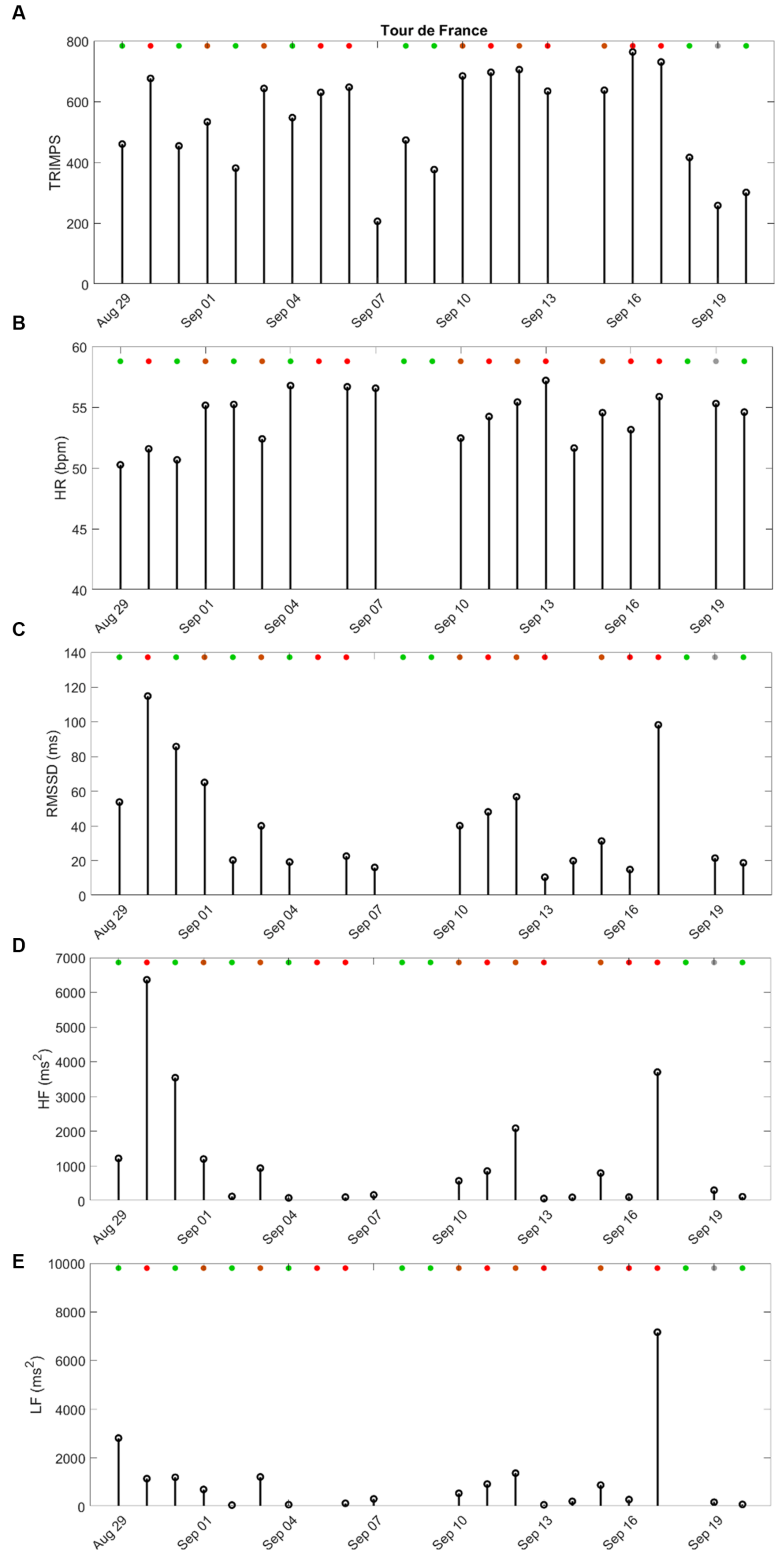
**(A)** TRIMPS (au) of Tour de France stages. **(B)** HR (bpm) in the supine position. **(C)** RMSSD (ms) in the supine position. **(D)** LF (ms^2^) in the supine position. **(E)** HF (ms^2^) in the supine position. All panelists during the Tour de France 2020 (29 August–20 September 2020). Green dots for flat stages, brown dots for rolling stages, red dots for mountain stages, and grey dots for time trial stage. No dot for resting days. No HRV values indicate that no HRV recording was performed on this day. LHTL, live high-train low-altitude training camp; LHTH, live high-train high-altitude training camp.

HRV and TRIMPS stage by stage during the Tour de France are reported in Figure 2. As expected, TRIMPSs were very high except for the recovery days and a few flat stages (Figure 2A). Supine HR remained high throughout the competition (Figure 2B). RMSSD and HF showed some recovery after resting days and flat stages (Figures 2C,D) but were generally low, whereas LF was low throughout the competition (Figure 2E).

CV is reported for each bloc along with TRIMPS and HRV values in Table 1. CV was lower for HR but higher for RMSSD, LF, and HF during the competitions than during the training period. These results must be put into perspective with the fact that there is a 2-fold decrease in RMSSD, a 4-fold decrease in LF, and a 3-fold decrease in HF in the two competition blocks.

**Table 1:** HRV in the supine position and TRIMPS over the entire period and during competition block #1 and the Tour de France, respectively.

	Overall 14 May–01 October	Training 14 May–05 August	Competition block #1 06 August–23 August	Tour de France 29 August–20 September
TRIMPS (a.u.)	403 ± 226	353 ± 230	534 ± 267 *	550 ± 157 *
HR (bpm)	Mean 49 SD 5 CV 9%	Mean 47 SD 4 CV 9%	Mean 50 * SD 3 * CV 5% *	Mean 54 *^$^ SD 2 * CV 4% *
RMSSD (ms)	Mean 78 SD 35 CV: 47%	Mean 96 SD 26 CV 26%	Mean 48 * SD 26 CV 56% *	Mean 46 * SD 30 CV 72% *
HF (ms^2^)	Mean 2,769 SD 2125 CV: 78%	Mean 3,590 SD 1858 CV: 58%	Mean 1,320 * SD 1446 CV: 107% *	Mean 1,267 * SD 1683 CV: 141% *
LF (ms^2^)	Mean 2,431 SD 2640 CV: 107%	Mean 3,319 SD 2819 CV: 85%	Mean 798 * SD 733 * CV: 90%	Mean 1,097 * SD 1657 * CV: 160% *

TRIMPS, training impulse; a.u., arbitrary unit; HR, heart rate; bpm, beat per minute; RMSSD, root mean square of the successive differences; HF, high frequency; LF, low frequency; SD, standard deviation; CV, coefficient of variation. *Different from training. $Different from block #1.

## Discussion

This case study reports HRV values and training loads during an entire season for one of the best professional cyclists, including during competition. First, it confirmed the relationship between training loads and autonomic responses throughout the season. Second, since LF, HF, and RMSSD were low (and HR was high) during competition periods, there were no relationships between the high loads during the multi-day races and the HRV responses. However, the LF/HF ratio increased during the Tour de France, while it remained steady (and slightly above 1) during competition block #1.

### HRV during the training period

The present data confirm previous reports in Olympic champions (Plews et al., 2012; Schmitt et al., 2016), where HRV was modulated in response to variations in training loads. The strong influence of altitude training camps on HR and HRV was also confirmed. During the second half of July, LF kept increasing, while RMSSD and HF started to decrease, which put the athlete in a situation of sympathetic hypertonia (as seen on Figure 2F with the increase in LF/HF ratio), likely preceding latent fatigue (Schmitt et al., 2015), which was adequately counterbalanced by a recovery period (i.e., a decrease in TRIMPS).

### Altered HRV during the competition periods

During competition, there is a 4-fold decrease in LF, a 3-fold decrease in HF, and a 4 bpm increase in HR (Table 1). Such alterations in HRV have been associated with overload and potential decreased performances in athletes (Schmitt et al., 2015). Despite the enormous load during competition periods, this athlete kept performing and finished in the top 12 of the Tour de France, likely moving from a trained status (high LF and HF and low HR) to a functionally overreached status (low LF and HF and high HR). It is likely that if the competition period extends without adequate recovery, it will lead to non-functional overreaching or overtraining syndrome (Meeusen et al., 2013). Accordingly, the LF/HF ratio did not drastically change during competition block #1 and increased during the Tour de France, yet it remained within the range of values observed during pre-season training.

### Analysis of the coefficient of variation

On the one hand, the coefficient of variation varied according to previous findings for HR (Plews et al., 2012), showing a significant decrease from the training period to the two competition blocks, while the average HR significantly increased by 7 bpm (14%) from the training period to the Tour de France. On the other hand, the CV significantly increased for RMSSD, LF, and HF, which is contrary to previous reports (Plews et al., 2012). Remarkably, the load was so high during the competition blocks that there was a 2-fold decrease in RMSSD, a 4-fold decrease in LF, and a 3-fold decrease in HF, which mathematically resulted in the significant increases in CV reported in Table 1. This novel and interesting observation suggests that the CV that was shown to be a valid marker when the average of a parameter does not drastically change from one time point to another (Plews et al., 2012) (i.e., in athletes who do not have competition lasting several weeks) may not be valid when a 2–4-fold decrease occurs. In this latter case, the interpretation of CV is problematic.

### Practical implication

In the current study, competition periods corresponded to an increase of 63% in TRIMPS, which resulted in altered HRV, yet the athlete’s performance remained good as he finished as top 3 on the Critérium du Dauphiné and top 12 for his fourth participation in the Tour de France (and best ranking at this time). Although the reported HRV responses during competitions were of interest as a marker of the extreme load induced by multi-stage competitions (Meeusen et al., 2013), one may question the relevance of such daily recordings since there is no possibility for the athlete or the coach to modify the training loads dictated by the stage characteristics (distance and elevation) and the race intensity. The only possible means of adaptation would be to amend the athlete’s participation in the next competition.

## Limitations

In this study, recordings in the supine position were only available for practical reasons. In the morning before the races, elite cyclists’ available time is very limited; therefore, adding an orthostatic stressor (e.g., standing position) was too time-consuming and may have had a negative impact on the athlete (they often report a sensation of discomfort during the standing position). Yet, adding an orthostatic stressor would have likely allowed for better capture of the athlete’s response to training and races (Schmitt et al., 2015).

During a multi-stage race such as the Critérium du Dauphiné or the Tour de France, the cyclist sleeps in a new hotel every night. Therefore, it is likely that the changed conditions (e.g., hotel room and temperature) increase the inter-day variability in the HRV responses. In addition, although the cyclist was closely followed by the team nutritionist and the team doctor for hydration and medication, respectively, these factors were not accurately measured. However, we strongly believe that the large 3–4-fold decrease in HRV during competition periods was mainly caused by the extreme loads and only marginally impacted by other factors such as nutrition, hydration, or medication.

## Conclusion

This case study reports altered HRV responses during elite cycling competitions. The reported alterations in HRV were compatible with previously characterized fatigue profiles (Schmitt et al., 2015) despite the athlete’s performance remaining his best in competition (best personal performance on the Tour de France), indicating potential functional overreaching but no non-functional overreaching. Several physiological differences have been reported between athletes with functional overreaching or non-functional overreaching (e.g., decrease in peak lactate or maximal cardiac output, change in catecholamines, and alteration in mood and/or self-confidence) (Le Meur et al., 2013; Bellenger et al., 2021). The limitations of such measurements daily confirm that HRV follow-up is relevant and one of the most practical means during training periods, but question its clinical usefulness during multi-stage cycling competitions, where complementary recordings as subjective fatigue scales may be more appropriate.

## Data availability statement

The raw data supporting the conclusions of this article will be made available by the authors, without undue reservation.

## Ethics statement

The studies involving humans were approved by Necker Hospital Ethics Committee (Paris, France). The studies were conducted in accordance with the local legislation and institutional requirements. The participants provided their written informed consent to participate in this study. Written informed consent was obtained from the individual(s) for the publication of any potentially identifiable images or data included in this article.

## Author contributions

NB and GM designed the study and drafted the manuscript. SB recorded the data. NB and LS analyzed the data. NB prepared the figures. All authors approved the final version of the manuscript.
